# Natural products and drug discovery: a survey of stakeholders in industry and academia

**DOI:** 10.3389/fphar.2015.00237

**Published:** 2015-10-26

**Authors:** Vafa Amirkia, Michael Heinrich

**Affiliations:** Centre for Pharmacognosy and Phytotherapy/Research Cluster Biodiversity and Medicines, UCL School of Pharmacy, University College LondonLondon, UK

**Keywords:** natural products, drug discovery, academia-industry links, Big Pharma, HTS, strategy

## Abstract

**Context:** In recent decades, natural products have undisputedly played a leading role in the development of novel medicines. Yet, trends in the pharmaceutical industry at the level of research investments indicate that natural product research is neither prioritized nor perceived as fruitful in drug discovery programmes as compared with incremental structural modifications and large volume HTS screening of synthetics.

**Aim:** We seek to understand this phenomenon through insights from highly experienced natural product experts in industry and academia.

**Method:** We conducted a survey including a series of qualitative and quantitative questions related to current insights and prospective developments in natural product drug development. The survey was completed by a cross-section of 52 respondents in industry and academia.

**Results:** One recurrent theme is the dissonance between the perceived high potential of NP as drug leads among individuals and the survey participants' assessment of the overall industry and/or company level strategies and their success. The study's industry and academic respondents did not perceive current discovery efforts as more effective as compared with previous decades, yet industry contacts perceived higher hit rates in HTS efforts as compared with academic respondents. Surprisingly, many industry contacts were highly critical to prevalent company and industry-wide drug discovery strategies indicating a high level of dissatisfaction *within* the industry.

**Conclusions:** These findings support the notion that there is an increasing gap in perception between the effectiveness of well established, commercially widespread drug discovery strategies between those working in industry and academic experts. This research seeks to shed light on this gap and aid in furthering natural product discovery endeavors through an analysis of current bottlenecks in industry drug discovery programmes.

## Introduction

Historically, natural products (NP) development has been a field of immense interest to medical, commercial, and scientific communities worldwide. As isolation and purification techniques advanced, NP increasingly became prime candidates for drug leads and drug discovery efforts (Cragg et al., 1997; Heinrich and Gibbons, 2001; Heinrich, 2013). Their diversity characterizes them as a virtually limitless source of novel lead compounds.

Yet in the last decade, the majority of multinational pharmaceutical companies have reduced NP Research and Development (R&D) expenditures (David et al., 2014). Many of the largest pharmaceutical companies are aggressively downsizing internal scientific research teams and reducing cost/risk by focusing instead on acquisitions of SMEs, which do the bulk of discovery “legwork” for a particular compound as it gets pushed through the pipeline.

What are elements behind this? What are the common drivers and barriers in natural product development? How can efforts to understand such drivers and barriers (Amirkia and Heinrich, 2014) enhance our ability to further leverage the potential of NPs? If NPs have historically been such an important source of new medicines, what insights can we gain into the NP drug development process of academic stakeholders as compared with the widely recognized slowdown of industry efforts? What are the differences in insights between successes of NP drug discovery today among in industry and academia? Through this research, the authors seek to gain insight into these questions by directly soliciting the views of an unprecedentedly large panel of pharmaceutical industry experts who *currently serve* in senior positions in academic/commercial organizations. To be best of our knowledge, it is the first published survey of stakeholders in the NP drug discovery sector.

## Backgorund: Current context of drivers and barriers in natural product development

A brief but representative selection of NP development and drug discovery-related opinion, review, and primary literature published over the last two decades shows a range of varied, often contrasting viewpoints on the potential of NPs as drug leads/candidates (Table 1). The majority of published literature hails the potential of NPs as sources of structurally novel, highly diverse compounds and cites examples of how NPs comprise a high proportion of successfully marketed new medicines over the last 20 years. The voice of optimism is loud and clear and has generally overshadowed a number of critical voices which have pointed out major challenges in NP development such as extraction and supply issues (McChesney et al., 2007). Additionally, many have added fuel to this debate through focusing on academia-industry partnership initiatives, inter-disciplinary approaches such as virtual screening methods and genomics efforts; one example being Shen's paper in 2003 which outlined three main advantages of virtual screening of natural products. He argued that virtual screening provides: higher hit rates as compared with typical HTS assays thus saving time/cost. Additionally he considers it to be more effective in investigating the about 90% of the “natural diversity” which so far have not been explored (defined as species which have yet to be studied systematically in research settings), and increased prediction of ADME/Tox and other drug like properties which may show promise in diminishing missed/failed hits (Shen et al., 2003; Bohlin et al., 2010).

**Table 1:** **Selection of representative publications on the outlook of NPs as drug leads in modern drug discovery programs and their overall levels of optimism**.

**Title**	**Author and year of publication**	General outlook/Tone^*^
Recent natural products based drug development: a pharmaceutical industry perspective	Shu, 1998	Optimistic
Natural product drug discovery in the next millennium	Cragg and Newman, 2001	Optimistic
Natural products in the process of finding new drug candidates	Vuorelaa et al., 2004	Optimistic
The role of natural product chemistry in drug discovery	Butler, 2004	Neutral
The renaissance of natural products as drug candidates	Paterson and Anderson, 2005	Optimistic
Drug discovery from medicinal plants	Balunas and Kinghorn, 2005	Optimistic
The evolving role of natural products in drug discovery	Koehn and Carter, 2005	Optimistic
Drug discovery from natural products	Gullo et al., 2006	Optimistic
Drug discovery from natural sources	Chin et al., 2006	Optimistic
Plant natural products: back to the future or into extinction?	McChesney et al., 2007	Pessimistic
Challenges and opportunities in drug discovery from plants	Jachak and Saklani, 2007	Optimistic
A review of high throughput technology for the screening of natural products	Mishra et al., 2008	Neutral
New aspects of natural products in drug discovery	Lam, 2007	Neutral
The value of natural products to future pharmaceutical discovery	Baker et al., 2007	Neutral/Pessimistic
Molecular understanding and modern application of traditional medicines: triumphs and trials	Corson and Crews, 2007	Neutral/Optimistic
Natural products in drug discovery	Harvey, 2008	Optimistic
Natural products as a robust source of new drugs and drug leads: past successes and present day issues	Rishton, 2008	Neutral
Drug discovery and natural products: end of an era or an endless frontier?	Li and Vederas, 2009	Neutral
Modern natural products drug discovery and its relevance to biodiversity conservation	Kingston, 2010	Optimistic
The impact of the united nations convention on biological diversity on natural products research	Cragg et al., 2012	Neutral
The pharmaceutical industry and natural products: historical status and new trends	David et al., 2014	Neutral
The re-emergence of natural products for drug discovery in the genomics era	Harvey et al., 2015	Optimistic

* *The general outlook the potential contribution of natural products in modern drug discovery efforts is summarized as pessimistic, neutral, and/or positive. Clearly the overall optimism in published assessment contrasts with the current trends in R&D activities in the pharmaceutical industry*.

One limitation of many if not all of these studies is that in essence they are based either on an assessment of new drug (leads) or are basically opinion papers. The authors normally had not engaged with a substantial number of stakeholders from *within* the pharmaceutical industry; most importantly those who *currently* work in the industry. Understandably so, not only is it challenging to track down a meaningful number of industry decision makers with experience in NP drug development but perhaps the larger challenge is eliciting their views (often which are critical of their superiors) pertaining to their company's strategy and/or industry trends. The authors believe that this internal lens, through angles such as commercial operations, strategic planning, research and development, and senior management is essential in gaining a clearer understanding of the role of NP discovery and development as it contributes to drug development in general, as well as the gaps, and potential advances in academia-industry partnerships to advance drug discovery efforts.

## Methods: Engaging industry contacts

A panel of industry and academic contacts (most of which are personal contacts of one or both of the authors) were personally invited to participate and submit insights to a natural products development survey which was hosted online (Google Forms—http://forms.google.com). A snowballing strategy was used to increase the number of contacts. Industry contacts represented many of the major multinational pharmaceutical companies such as Merck, Novartis, GSK, Pfizer, AZ, and Bayer. Seniority of each respondent varied with respect to his or her organization. Titles of respondents included: Chief Scientific Officer (CSO), President, Vice President (VP), Group Leader, Senior Analytical Chemist, and Senior Principal Scientist (Appendix 1 in Supplementary Material) among others.

Academic contacts originated from eight different countries including Brazil, Oman, New Zealand, UK, and USA. The majority of academic respondents were full-time academics, five of which also hold senior roles in pharmaceutical-company related organizations (consultancy, clinical research, and/or pharmaceutical entities). The panel is clearly limited in its geographical coverage of smaller pharmaceutical markets such as Asia, Japan, and Latin America; markets which represented approximately 11, 9, and 5% of 2014 total worldwide pharmaceutical sales, respectively (IMS Health, 2014). Nevertheless, barring the extreme of labeling the panel as strictly representative of “the industry,” the authors feel that the panel of contacts is generally representative of trends of interest within the industry.

There were four primary goals which were considered in designing the questionnaire:

1. To understand perceived drivers and barriers in NP drug discovery efforts.
2. To understand what respondents identify as “*current preferred strategies”* for discovering new medicines in industry today.
3. As HTS stands as a prevalent tool in in drug discovery today we wanted to elicit *perceptions of the efficacy of NPs* as compared with other classes of compounds in screens.
4. To understand the respondent's general outlook on future drug discovery as a whole. This approach would allow the authors to better understand the perceived effectiveness of past, present, and future NP drug discovery efforts and more importantly compare any potential similarities and differences in insights between academic and industry respondents.

The survey consisted of a series of six quantitative and qualitative close-ended questions followed by 10 profile and background related questions. Close-ended questions with several choices had an “other” box for the respondent to fill in his/her response which allows for valuable straightforward feedback from respondents and helps overcome the limitations of extensive statistical analyses based of a small sample size. Multiple choice selections were displayed in randomized order for each survey so as to control for position bias in responses. Close-ended questions were comprised of:

1. In your opinion, what are the top 2 current preferred strategies for drug discovery?
2. Based on your experience or on your assessment, approximately how many agents based on natural products and alkaloids researched in commercial R&D facilities make it to market as pharmaceutical products?
3. From your experience, what have been the major drivers to natural product development in industry?
4. From your experience, what have been the major barriers to natural product development in industry?
5. Drug Discovery is a history of triumphs and failures. Compared to last decades how successful is the industry today in discovering new medicines?
6. What is your outlook on the future viability (rate at which pharmaceuticals are developed and launched to market) of natural products, serving either as final pharmaceutical products or as leads to the development of the final pharmaceutical products?

The six close-ended questions each had an open field for participants to provide additional thoughts. Total completions of the survey ended at 52 responses after 14 weeks spanning from January to May 2015.

## Results and discussion

### Overview of findings

One major, consistent theme across respondents was the dissonance between what survey participants in industry perceived as the potential of NP as drug leads and overall industry and/or company level strategies. Large scale, structural modification processes (i.e., HTS) have become Big Pharma's go-to-strategy for honing in on successful leads. HTS typically avoids the need to continuously source and verify new NP material, which matches the highest citied barrier from industry contacts in our survey (i.e., a secure supply). Additionally, large HTS screening programmes are argued by many (Macarron et al., 2011) to be more cost-effective in the long run which is also in line with the third largest barrier cited by our industry contacts (i.e., cost/funding/budget). Sample sizes of respondent groups are not sufficiently large enough to perform useful statistical analysis yet general results are summarized below (Table 2).

**Table 2:** **Results of the survey including selected close ended and profile questions**.

	**Industry respondents (*n* = 33)**	**Academic respondents (*n* = 19)**
Average age	53	48
Average years of experience in the pharmaceutical industry	25	10
Male/Female	82% Male, 18% Female	89% Male, 11% Female
Drug Discovery is a history of triumphs and failures. Compared to last decades how successful is the industry today in discovering new medicines?	3.9^*^ (SD:1.22)	4.3^*^ (SD:1.37)
In your opinion, what are the top current preferred strategies for drug discovery?	High throughput screening (HTS)—31% Physiochemical—modifications to existing leads—25% Virtual/Computational prospecting/modeling—19%	High throughput screening (HTS)—34% Physiochemical—modifications to existing leads—24% Virtual/Computational prospecting/modeling—18%
Top **Drivers** to natural product development in industry^1^	Structural Novelty and Bioactivity—47% Efficacy and/or chemical viability (solubility, stability, toxicity, etc.)—18% Supply—11%	Structural Novelty and Bioactivity—42% Efficacy and/or chemical viability (solubility, stability, toxicity, etc.)—24% Cost/Funding/Budget—15%
Top **Barriers** to natural product development in industry^1^	Supply—26% Structural Complexity—20% Cost/Funding/Budget—19%	Cost/Funding/Budget—25% Structural Complexity—23% Supply—25%
What is your outlook on the future viability (rate at which pharmaceuticals are developed and launched to market) of natural products, serving either as final pharmaceutical products or as leads to the development of the final pharmaceutical products?^2^	Optimistic—52% Unsure/“Hard to say”—21% Pessimistic—27%	Optimistic—63% Unsure/“Hard to say”—26% Pessimistic—11%

* *Respondents were asked to rank the success of natural product based drug discovery efforts from 1 = Full of Triumph to 7 = Full of Failure. The values given are the averages from all responses*.

Two other major themes emerged from participants writing insights in the open space provided after each close-ended question and are illustrated with a selection of verbatim statements pertinent to each theme.

#### Ineffectiveness of current HTS drug discovery programmes

Industry efforts which boast large libraries and cutting edge screening technologies have gained momentum which in turn has overshadowed smaller, more unique and fruitful discovery efforts.

- “The industry focus on numbers (quantity vs. quality) has counted against natural products discovery—and the belief that supply of material on a suitable scale might be difficult (which may be a misconception).”
- “Industry is driven by numbers and processes; HTS, you could include fragment screening in this too—or even billions of compounds on encoded libraries (as we have at [X] company). I am part of a group who strongly advocate the huge impact of proper attention to physical properties and efficiency as existing leads are optimized (sadly mostly derived from the numbers generated above). HTS yet is the adopted strategy; in my opinion is probably isn't the most preferred!”
- “These approaches [High throughput screening (HTS), Combinatorial Chemistry] are favored by many pharmaceutical companies, even though they have not been notably successful.”
- “HTS depends on large libraries, most of which have been so thoroughly studied that their utility going forward must be considered modest.”
- “My understanding is that physicochemical modifications of existing leads represents the vast majority of drug discovery, and there are few places which are supporting anything beyond HTS or medicinal chemistry cycles.”
- *“Based on our internal track record, the outcome of HTS or VS is heavily dependent on the quality (control) of the actives and their ligand efficiency and the access to orthogonal assays to confirm the activity. These methods also complement each other and can be supported by additional methods, e.g., fragment-based. They are also generally easier to strip to the ‘core’ and obtain initial SAR. With natural products, you need to be lucky with the minor metabolites yielding some useful SAR*.

*Nevertheless, our experience at X University…screening endogenous X species was successfully generating leads that were NOT pursued as chemists perceived the SAR work to have low feasibility.”*

- *“In industry, modification of existing structures whether already in-house identified compounds or to bypass other structures with patent protection is much more common. This allows for the creation of “me too” therapeutic agents. Bioprospecting is much more common in academia, but natural product identification seems to be decreasing on the whole. Whether this is purely due to funding issues or a broader shift in the field is not certain. Similarly, virtual/computation approaches are used in refining structure in industry but are essentially never used to de novo identify a drug*.

*“Many academic labs have used such strategies as well, but with few successes. HTS is still fairly common place in industry and is gaining greater traction in academic settings with more and more universities creating screening facilities. Serendipity is certainly an important part of drug discovery, especially in areas such as neurology, but no one would bet on winning the lottery to fund their lab.”*

- *“HTS has been an abject failure in terms of discovery, due in most cases to not thinking about transfer across membrane issues when trying to go from a hit to an active in cells/animals*.

*If one uses phenotypic screening (a dirty term amongst screeners in Pharma!), then if you see a valid effect, you will be well ahead of any HTS assay in vitro.”*

- “In Pharma, HTS is the buzzword. I know of screens where over 1 million synthetic compounds have produced nothing, many times. Natural products in phenotypic screens are between 10,000 and 100,000 depending upon what is known about potential mechanisms etc.”

#### The second key theme that emerged centers around the lack of support/interest in organization for NP drug development efforts

Industry strategy over the last few decades has taken its form against a NP-centric strategy and is unlikely to change.

- “Don't fit company strategy.”
- “Executive management fiat. Senior and executive scientific management at most Big Pharma wrote off natural products in the late ‘80s and early ‘90s with the advent of HTS, believing that HTS would have all of the answers.”
- “Hostility; No support.”
- “Lack of will to study them.”
- “Natural product discovery tends to require a group to champion the approach. In my experience med chemists don't switch between synthetic chemistry and natural product chemistry. The latter requires an infrastructure and senior champions who believe in the potential of the approach. The novelty of the structures that result often go beyond anything that a med chemist might consider synthesizing as such this can take you to places you wouldn't have got to by any other route.”
- “Screening of synthetic chemicals in massive libraries is cheap and most often results in hits that can be optimized as leads effective against sign targets. This process discounts any deep understanding of the biological processes involved in a disease state, other than the role played by an individual target biomolecule (kinases, etc.). And, the chemistry involved in elaborating these often simple structures is easy and high throughput—so from the chemists standpoint—why knock yourself out with NP modifications which are often more difficult? Regrettably in industry little credit is given for the extra effort and overall productivity will appear low.”
- “The major driver to natural product development in industry is to eliminate it, which is what most of the large pharma companies have in fact done.”
- “From my perspective, today, natural products make only sense as starting materials for further optimization. I am convinced that we will see less and less original natural products that make it to the market in human pharma (animal health may be a different story). Also TCM et al. may be a different story.”
- “NPs are currently not the “flavor of the month or decade” but now days, chemists are looking for structural leads that may well have activity, due to the failure of combi-chem as a discovery tool.”

It is interesting to note that such an open question in fact only elucidated two key reasons why natural products are poorly represented in such drug discovery processes. Open questions are often used to elicit a wider set of views (Heinrich et al., 2009) and here a clear focus on two concerns emerged indicating a very strong consensus on that these are seen as the key issues.

### Perceived viability of natural products among current drug discovery programmes

Gaining insights into perceptions of the drivers and barriers of NP drug discovery is a helpful yet limited step in providing insights into the drug development process. This data does not convincingly indicate the “effectiveness” of NPs in drug discovery as compared to other commonly researched classes of compounds. Thus, we asked respondents to provide an approximate ratio of “success rates” for several classes of compounds in the following way: *Based on your experience or on your assessment, approximately how many synthetic [or Biologics, Natural Products, Alkaloids] agents researched in commercial R&D facilities make it to market as pharmaceutical products?* This resulted in four sets of answers relevant for each of these groups.

Interestingly, all perceived “hit rates” for industry respondents are higher than those academic respondents reported (Table 3). Industry respondent “hit rates” were higher at a rate ranging between 1.8 and 3.1-times. This may reaffirm our other finding that in general, senior stakeholders in industry typically do support NP-centric discovery strategies, and hence the more perceived frequent “hits.” Conversely, this indicates that screening programmes related to academic efforts, particularly with respect to HTS, are not *perceived* as being as useful more widespread industrial efforts. Does this mean that industry is more “productive” than academia in screening for natural products? Not necessarily, as this question does not attempt to equalize all screening methods but rather gain a general indication of respondent's perceptions toward screening efforts. Additionally, it is also surprising to note that there is a larger gap between “hit rates” reported between NPs and synthetics for industry vs. academic respondents; 8-times vs. 5-times, respectively. This also indicates a reaffirmation to our previous observation that many working in industry—regardless of their role and their level of dissatisfaction with the strategic direction of their organization, still perceive strong relative potential in NP drug development as compared with currently prevalent synthetic-centric strategies.

**Table 3:** **Respondent's estimates of how many agents researched commercial R&D facilities make it to market as pharmaceutical products (defined as the “hit rate”)**.

**Compound Class**	**Industry respondents (*n* = 33) (Logarithmic value)**	**Academia respondents (*n* = 19) (Logarithmic value)**
All natural products^*^	4.03 (1 in 10,723)	4.53 (1 in 33,598)
Alkaloids	4.27 (1 in 18,738)	4.53 (1 in 33,598)
Synthetic compounds	4.97 (1 in 93,260)	5.26 (1 in 183,298)
Biologics	3.67 (1 in 4642)	4.11 (1 in 12,743)
Overall average	4.24 (1 in 17,179)	4.61 (1 in 40,504)

*Respondents selected from a range of six responses beginning at “1 in 100” and ending at “1 in 1,000,000+.” Averages were calculated by assigning a value between 2 and 7 to each response and extrapolating through a logarithmic calculation (e.g., 10^2^ = 100, 10^6^ = 1,000,000)*.

* *All natural products includes alkaloids*.

Our goal in asking this question is two-fold; to gain a general indicator of perceived “success/hit rates” of NPs against other compound classes as well as compare the perception of “success rates” against previous claims published over the years by industry observers (Shen et al., 2003). There are two limitations to this question. The first is that each respondent may define “researched” in a completely differing way. To one respondent a compound is not “researched” until it perhaps enters a HTS program, while to another, a compound merely existing in a company compound library may count as being “researched.” The second is the definition of the compound class (for example: Where does a NP which has been structurally modified fit?). Of course, there are numerous variables in any screen (compound library itself, target/ligands, parameters for defining a successful “hit,” purpose of screen, etc…) that make a particular screen entirely unique and incomparable to another.

### Potential steps forward

Since 35 of the 52 *respondents* (35 of all 105 *individual answers* to this open question) listed HTS as a “top preferred current strategy,” it is logical that this should be a focus of our analysis. Many publications have cited barriers to NP drug development. In 2004 Jean-Yves Ortholand, who at the time worked at Merck in France, listed six major drawbacks in programmes screening natural products: expense, time, novelty, tractability, scale-up, and intellectual property (Ortholand and Ganesan, 2004). In looking to our industry feedback, each of Ortholand's “drawbacks” are corroborated to some extent with a particular focus on supply and cost/funding. It is noteworthy that these two highly cited barriers do not directly involve the actual screen itself but rather affect the feasibility of pre/post-screen efforts. The most frequently cited barriers seem to be those which prevent a screen from happening in the first place (i.e., budget/cost or company strategy) or from moving from early stage screening to pre-clinical development (i.e., supply, scale-up). Therefore, besides proposing the obvious that costs should be reduced and/or funding increased for NP drug discovery efforts, are there potential cost-sensitive resolutions to the supply/scale-up barrier?

Our previous research (Amirkia and Heinrich, 2014) looked at the problems of supply in the context of source species abundance data of pharmaceutical alkaloids. We showed that source species of pharmaceutical alkaloids are on average 4.3 times more “abundant” [as defined by the Global Biodiversity Information Facility (GBIF) species abundance dataset] than a randomly picked non-pharmaceutical alkaloid. Alkaloid containing species yielding medicines are thus much more widely distributed than species which yield alkaloids not used pharmaceutically. This suggests that such a dataset is sufficiently significant for modeling supply constraints which are so often cited in NP related literature. Although this initial analysis was performed on alkaloids, it can be applied to any class of NPs.

Taken together our data show that such an analysis can be augmented with other metrics such as number of countries which host species naturally occur in or density of source species abundance (Amirkia and Heinrich, 2014). Here key questions include:

- Is the source species widely spread across one region or densely found in one small area?
- How many countries does the species naturally grow in?
- How are occurrences of the species in the dataset distributed across time? Were instances discovered and recorded decades ago or have records been relatively consistent?

Instead a systematic assessment of a species' abundance can play a constructive pre-screening or filtration role in NP drug discovery programmes which directly addresses one of the key concerns of the stakeholders who contributed to this study. Costs for such analyses are minimal compared to R&D budgets common to pharmaceutical companies today. Additionally such analyses need not necessarily be exclusively seen as applicable to screening programmes for candidates or leads but may prove to be of value in other NP-related endeavors. For example, companies which are heavily invested or interested in TCM, Ayurveda, and other traditional medicine centric portfolios may use this approach to optimize procurement or investment processes. Compounds which originate from source species which are becoming increasingly abundant may hold more promise long-term sustainability in production and marketability.

## Conclusions

NPs are seen as important sources of new medicines by industry stakeholders, yet, the industry is spending fewer and fewer resources on their discovery and development. In the last decade numerous voices have highlighted this concern with the vast majority of these originating from academic and industry observers (Niedergassel and Leker, 2009; Tralau-Stewart et al., 2009; Khanna, 2012). Two thirds of panel responses cited HTS as the preferred strategy for drug discovery in industry today and NPs are seen as yielding higher “hit rates,” thus industry attention must not turn away from NPs if the industry seeks innovation. This gap must be explored if we are to move natural product drug discovery forward and virtual non-ligand machine learning, at least for alkaloids, can serve as a starting point to guide multidisciplinary drug discovery efforts.

While this study has some limitations both in terms of the overall size of the sample and the (self-) selection of participants, the voices from within clearly highlight some key concerns, which can be overcome by implementing a modified strategy in NP-driven drug development. Minimizing one of the most commonly cited barriers (i.e., supply) by seeking to quantifying it and developing strategies for incorporating solutions at an early stage of screening programs is one approach which has demonstrated promise for alkaloids and can be applied to other NP drug discovery efforts. The work also strengthens the case that “weeds” are an important source of drugs (Stepp, 2004), but offers a quantifiable parameter to assess such “weediness.” Continuing along the current path of large “numbers-driven” screens which boast millions of compounds not only has irritated many, at all levels, in the industry but more importantly stifled the growth and development of the single most productive source of potential leads for new medicines to date; nature.

### Conflict of interest statement

The authors declare that the research was conducted in the absence of any commercial or financial relationships that could be construed as a potential conflict of interest.

## Footnotes

^1^Respondents could select or list more than one response. All responses were added together and a “response rate” was calculated by taking the percentage of a particular response as a total of all responses.

^2^Respondents selected one response. All responses were added together and a “response rate” was calculated by taking the percentage of a particular response as a total of all responses.

## Abbreviations

GBIF: Global Biodiversity Information Facility
HTS: high throughput screening
NP: natural products
SAR: structure-activity relationships
SD: standard deviation
SME: small medium enterprise
VS: virtual screening.
