# Supplementary material for: Natural products and drug discovery: a survey of stakeholders in industry and academia
Source: Front Pharmacol. 2015 Oct 26;6:237. doi: 10.3389/fphar.2015.00237 (PMC4620409; doi:10.3389/fphar.2015.00237)
Supplement: Supplementary file 1 [file DataSheet1.DOCX]

**Appendix**

| Age | Highest Educational Degree | Years of pharmaceutical industry exp. | Which country are you currently based in? | Size of the institution you belong to | Your title: | Knowledge natural product development comes from: |
| --- | --- | --- | --- | --- | --- | --- |
| 65 | PhD / MD / PharmD / Doctorate | 35 | USA | 11-100 | CSO | Industry (Research) |
| 58 | PhD / MD / PharmD / Doctorate | 25 | USA | 1-10 | President | Industry (Research) |
| 67 | PhD / MD / PharmD / Doctorate | 40 | UK | 1-10 | Director | Industry (Management) |
| 57 | PhD / MD / PharmD / Doctorate | 25 | UK | 999-10,000 | Supervisor | Academia |
| 67 | PhD / MD / PharmD / Doctorate | 40 | UK | 1-10 | Director | Industry (Management) |
| 52 | PhD / MD / PharmD / Doctorate | 26 | UK | 1-10 | Project Manager | Industry (Management) |
| 54 | PhD / MD / PharmD / Doctorate | 16 | UK | 10,000+ | Dr | Academia |
| 45 | PhD / MD / PharmD / Doctorate | 9 | USA | 10,000+ | Principal Scientist | Academia |
| 72 | PhD / MD / PharmD / Doctorate | 40 | USA | 10,000+ | Retired CSO | Industry (Research) |
| 75 | PhD / MD / PharmD / Doctorate | 37 | USA | 10,000+ | Retired | Industry (Research) |
| 70 | PhD / MD / PharmD / Doctorate | 42 | USA | 1-10 | Ph.D. | Industry (Research) |
| 59 | PhD / MD / PharmD / Doctorate | 35 | USA | 10,000+ | Research Fellow | Industry (Research) |
| 49 | PhD / MD / PharmD / Doctorate | 22 | Belgium | 10,000+ | Scientific Affairs | Industry (Management) |
| 42 | PhD / MD / PharmD / Doctorate | 13 | Switzerland | 10,000+ | Senior investigator | Industry (Research) |
| 76 | PhD / MD / PharmD / Doctorate | 0 | USA | 999-10,000 | Professor | Academia |
| 59 | PhD / MD / PharmD / Doctorate | 30 | Italy | 11-100 | Professor | Academia |
| 62 | Bachelors | 15 | USA | 1-10 | VP Analytical Chemistry | Academia |
| 56 | PhD / MD / PharmD / Doctorate | 30 | USA | 101-999 | Professor and Director | Academia |
| 75 | PhD / MD / PharmD / Doctorate | 25 | USA | 10,000+ | Retired Chief | Industry and Government |
| 39 | PhD / MD / PharmD / Doctorate | 0 | UK | 101-999 | Reader | Academia |
| 58 | PhD / MD / PharmD / Doctorate | 30 | Germany | 10,000+ | CVP | Industry (Research) |
| 53 | PhD / MD / PharmD / Doctorate | 21 | USA | 11-100 | Senior Scientist | Industry (Research) |
| 51 | PhD / MD / PharmD / Doctorate | 22 | USA | 10,000+ | Executive Director | Academia |
| 47 | PhD / MD / PharmD / Doctorate | 0 | USA | 11-100 | Sr. Research Scientist | Academia |
| 54 | PhD / MD / PharmD / Doctorate | 28 | USA | 10,000+ | Professor and Director | Industry (Research) |
| 34 | PhD / MD / PharmD / Doctorate | 10 | USA | 10,000+ | Managing Director | Industry (Research) |
| 46 | PhD / MD / PharmD / Doctorate | 15 | Germany | 10,000+ | Dr. | Industry (Research) |
| 40 | PhD / MD / PharmD / Doctorate | 12 | UK | 11-100 | Asso. Dir. of Discovery | Industry (Research) |
| 57 | PhD / MD / PharmD / Doctorate | 27 | USA | 10,000+ | Director | Industry (Management) |
| 55 | PhD / MD / PharmD / Doctorate | 25 | France | 999-10,000 | Dir. of Bot. and R&D Sourcing | Industry (Management) |
| 29 | PhD / MD / PharmD / Doctorate | 1 | USA | 999-10,000 | Research Scholar | Academia |
| 42 | PhD / MD / PharmD / Doctorate | 13 | Switzerland | 10,000+ | Director | Industry (Research) |
| 42 | PhD / MD / PharmD / Doctorate | 13 | Switzerland | 10,000+ | Senior Investigator | Industry (Regulatory) |
| 35 | PhD / MD / PharmD / Doctorate | 5 | USA | 999-10,000 | Research Scientist | Academia |
| 45 | PhD / MD / PharmD / Doctorate | 20 | India | 101-999 | Senior Manager | Industry (Research) |
| 39 | PhD / MD / PharmD / Doctorate | 0 | Oman | 11-100 | Associate professor | Academia |
| 50 | PhD / MD / PharmD / Doctorate | 10 | USA | 101-999 | Professor | Academia |
| 38 | PhD / MD / PharmD / Doctorate | 0 | New Zealand | 10,000+ | Senior Lecturer | Academia |
| 60 | PhD / MD / PharmD / Doctorate | 10 | USA | 10,000+ | CSO | Academia |
| 52 | PhD / MD / PharmD / Doctorate | 20 | Spain | 10,000+ | Director | Industry (Research) |
| 52 | PhD / MD / PharmD / Doctorate | 25 | UK | 10,000+ | Group Leader | Industry (Research) |
| 60 | PhD / MD / PharmD / Doctorate | 29 | USA | 10,000+ | Senior Principal Scientist | Industry (Research) |
| 40 | PhD / MD / PharmD / Doctorate | 10 | USA | 10,000+ | Chief Scientist | Industry (Research) |
| 62 | PhD / MD / PharmD / Doctorate | 40 | UK | 1-10 | CEO | Development |
| 40 | Masters | 18 | UK | 999-10,000 | Chief | Industry (Management) |
| 60 | PhD / MD / PharmD / Doctorate | 37 | Germany | 999-10,000 | VP | Industry (Management) |
| 53.8 | Avg. age | 20.6 | Avg. years of pharmaceutical industry exp. |  |  |  |

Appendix 1: Profile of selected respondents (46 of the total 52 respondents)
